# Magnesium sulfate gargle for preventing postoperative sore throat following laryngeal mask airway ventilation: a randomized, controlled, dose-finding trial

**DOI:** 10.3389/fphar.2026.1798716

**Published:** 2026-07-10

**Authors:** Lei Guo, Ling Ma, Shaoling Ma, Hanxiang Ma, Qiming Ma

**Affiliations:** Department of Anesthesiology and Perioperative Medicine, General Hospital of Ningxia Medical University, Yinchuan, China

**Keywords:** gargle, gynecological laparoscopic surgery, laryngeal mask airway, magnesium sulfate, postoperative sore throat

## Abstract

**Background:**

Magnesium sulfate gargle is widely used as an intervention for preventing postoperative sore throat (POST) due to its analgesic effect achieved by inhibiting calcium influx mediated by peripheral NMDA receptors. This study aimed to investigate its optimal dosage following laryngeal mask airway (LMA) ventilation.

**Methods:**

A total of 239 patients aged 18–60 years, with ASA grades I-II, who under general anesthesia with laryngeal mask airway intubation were included. Patients were randomized to gargle 30 ml of either normal saline or magnesium sulfate at 10, 20, or 30 mg/kg 10–15 min before anesthesia, with the primary outcome being the incidence of POST assessed in the PACU and at 2, 6, and 24 h postoperatively. The secondary outcomes included LMA ventilation-related parameters and adverse events.

**Results:**

The incidence of POST under different dosages of magnesium sulfate was 31.7%, 18.3%, 11.9%, and 6.7% in the PACU; 28.3%, 15.0%, 6.8%, and 6.7% at 2 h; 23.3%, 10.0%, 3.4%, and 3.3% at 6 h; and 15.0%, 5.0%, 3.4%, and 1.7% at 24 h postoperatively. As the dosage of magnesium sulfate gargle increased, the incidence of POST decreased (PACU, *P* = 0.002; 2 h postoperatively, *P* = 0.002; 6 h postoperatively, *P* < 0.001; 24 h postoperatively, *P* = 0.012). The ED90 values of magnesium sulfate gargle for preventing POST in the PACU and at 2 h postoperatively were 22.747 mg/kg (95% CI: 16.524–37.616) and 18.828 mg/kg (95% CI: 12.847–31.396), respectively. No differences were observed in LMA ventilation-related parameters and adverse events across groups.

**Conclusion:**

The prophylactic administration of magnesium sulfate gargle can effectively reduce the incidence of POST, with a dosage of 20 mg/kg identified as optimal.

## 1 Introduction

Postoperative sore throat (POST) is the most common complication of general anesthesia, with an incidence ranging from 26.5% to 76.9%, adversely affecting patients’ recovery experience and satisfaction; in severe cases, it may lead to dysphagia and dysarthria, thereby impairing postoperative recovery progress and potentially increasing both hospitalization duration and healthcare costs (Teshome et al., 2023; Mitobe et al., 2022; Moulder et al., 2016). Although supraglottic airway devices, such as laryngeal mask airway (LMA), are currently associated with a reduced incidence and severity of POST compared to tracheal intubation—primarily due to minimized exposure to laryngoscopy and mechanical irritation of the tracheal rings—their use may still result in pharyngeal compression and edema during gynecological laparoscopic surgery performed in the head-down position, thereby contributing to the development of POST (Farazmehr et al., 2021; Wang et al., 2025).

Magnesium sulfate, ketamine, and other NMDA receptor antagonists play a significant role in preventing POST, promoting wound healing, and improving epithelial tissue condition through anti-inflammatory and local analgesic effects (Mathew and Panonnummal, 2021; Kuriyama et al., 2020). Among these, magnesium sulfate gargle is widely regarded as a safe and effective intervention for preventing POST, as it achieves its analgesic effect by inhibiting calcium influx mediated by peripheral NMDA receptors, and its rapid ionization in solution enables easy absorption of magnesium (Mondal et al., 2023), with no evidence regarding the optimal gargle dosage. This study aims to investigate the optimal dosage of magnesium sulfate gargle for preventing POST following LMA ventilation in patients undergoing gynecological laparoscopic surgery under general anesthesia.

## 2 Methods

### 2.1 Trial design

This study was approved by the Ethics Committee of the General Hospital of Ningxia Medical University (Approval No. KYLL-2025–0376) and conducted in accordance with the Consolidated Standards of Reporting Trials (CONSORT) statement. The trial was carried out from March 2025 to October 2025 and adhered to the principles outlined in the Declaration of Helsinki. All patients provided written informed consent prior to enrollment. The study protocol was prospectively registered with the Chinese Clinical Trial Registry (ChiCTR2500097804) before the initiation of data collection.

### 2.2 Participants

Patients aged 18–60 years with American Society of Anesthesiologists (ASA) physical status I–II who underwent gynecological laparoscopic surgery under general anesthesia using a LMA ventilation were included. Exclusion criteria comprised preoperative chronic throat pain or inflammation; respiratory system disorders such as bronchitis, chronic obstructive pulmonary disease (COPD), and other related conditions; known hypersensitivity to or contraindications against magnesium sulfate; anticipated surgical duration of less than 1 h or more than 4 h; and suspected or anticipated difficult airway.

### 2.3 Intervention

The patient was transferred to the operating room and underwent non-invasive vital sign monitoring, which included electrocardiography (ECG), non-invasive systolic blood pressure (SBP), transcutaneous pulse oxygen saturation (SpO_2_), and respiratory rate (RR). An 18-gauge intravenous catheter was inserted in the upper limb for fluid and drug administration. Prior to general anesthesia induction, the patient was randomly assigned to either the normal saline (NS) group or one of three magnesium sulfate gargle dosage groups (10 mg/kg, 20 mg/kg, or 30 mg/kg), according to a computer-generated random number sequence with equal allocation (1:1:1:1 ratio) using SPSS software. A researcher who was not involved in the anesthesia management was responsible for disclosing the group allocation. The randomization schedule was stored in sealed, opaque envelopes. According to the allocated group, this researcher prepared magnesium sulfate solution with a total volume of 30 ml, which was unlabeled. Both the patients and the anesthesiologists remained blinded to the intervention assignment. All patients received the designated dose of magnesium sulfate gargle 10–15 min prior to the induction of general anesthesia. The gargle was administered by the anesthesiologists, who requested the patient to tilt their head backward and gargle while feeling the liquid at retropharynx, and the gargling duration was standardized to 30 s.

### 2.4 General anesthesia and monitor

General anesthesia was administered by anesthesiologists with more than 10 years of experience in tracheal intubation. All patients received standardized anesthesia management and perioperative nursing care. Preoperatively, patients were required to fast for 8 h and abstain from oral fluids for at least 4 h, with no additional premedication administered. Rapid sequence induction of general anesthesia was performed in all cases, using intravenous midazolam (0.05 mg/kg), sufentanil (0.4 μg/kg), etomidate (0.3 mg/kg), and rocuronium (0.8 mg/kg). Following 3 min of preoxygenation via face mask ventilation, a double-lumen LMA (Zhejiang Youyi Medical Technology Co., Ltd., China) was inserted. The appropriately sized LMA (3–4#) was selected based on the patient’s body weight and physique, and then lubricated thoroughly with medical-grade gel on its posterior surface before being inserted using a single-operator, two-hand blind technique.

Following intubation of the LMA, successful placement was confirmed when adequate ventilation was achieved without significant air leakage, bilateral chest rise was symmetrical, the end-tidal carbon dioxide (PetCO_2_) waveform displayed a clear expiratory plateau, no gas escapes from the mouth or nose, and peak airway pressure (Ppeak) remained within normal limits. During the insertion of the LMA, complete displacement from the airway constituted one intubation attempt. If the total duration of attempts exceeded 3 min or oxygen saturation (SpO_2_) decreased, the LMA should be removed immediately and manual ventilation with a face mask and positive pressure oxygenation should be resumed. A maximum of three intubation attempts was permitted; beyond this, tracheal intubation should be performed.

General anesthesia was maintained via continuous intravenous infusion of propofol at a rate of 4–6 mg/kg/h and remifentanil at 0.1–0.3 μg/kg/min. Rocuronium was administered intermittently to ensure adequate muscle relaxation. Tidal volume (VT) was set at 6–8 ml/kg, and respiratory parameters were adjusted as needed to maintain PetCO_2_ levels within the target range of 30–40 mmHg. All patients underwent bispectral index (BIS) monitoring to assess anesthetic depth, with BIS values maintained between 40 and 60. Propofol was infused accordingly to maintain an appropriate depth of anesthesia based on real-time BIS feedback. Following the restoration of spontaneous respiration and fulfillment of extubation criteria, the LMA was removed. Rocuronium-induced neuromuscular blockade was reversed using sugammadex at a dose of 1–2 mg/kg. The patient was subsequently transferred to the post-anesthesia care unit (PACU) for monitoring and recovery. Following the operation, the patient received patient-controlled intravenous analgesia (PCIA) consisting of sufentanil 2 μg/kg diluted with normal saline to a total volume of 100 mL, with an initial bolus of 1 μg/kg, a background maintenance rate of 2 mL/h, and a rescue bolus of 2 mL with a 15-min lock-out interval.

### 2.5 Outcomes

The primary outcome was the incidence of POST assessed in the PACU and at 2, 6, and 24 h postoperatively. Secondary outcomes included the severity of POST at the same time points, measured on a three-point scale (1 = mild, 2 = moderate, 3 = severe). Additional variables recorded were perioperative comorbidity, Mallampati classification, and type of surgery. LMA ventilation-related parameters included duration of intubation; LMA intubation time (defined as the duration from picking up the LMA to successful placement and connection to the breathing circuit, with adequate chest rise during manual ventilation and the first detectable PetCO_2_ waveform on monitoring confirming proper placement); LMA removal time (time elapsed from discontinuation of anesthetic agents to removal of the LMA); success on first attempt (proportion of cases with successful placement on the first attempt); intraoperative repositioning (proportion of required adjustments of LMA during surgery); conversion to tracheal intubation rate (proportion of conversion to endotracheal intubation due to inadequate ventilation); and oropharyngeal leak pressure (OLP). OLP was measured during manual ventilation by closing the expiratory valve of the anesthesia circuit, maintaining a fresh gas flow of 3 L/min, and recording the airway pressure at plateau (Yan et al., 2023). The measurement was terminated if audible oropharyngeal air leakage occurred, or if OLP exceeded 35 cmH_2_O, or SpO_2_ dropped below 95%. Adverse events included blood staining on the LMA, cough, hypoxemia (SpO_2_ < 90%), hoarseness, and dysphagia.

### 2.6 Sample size calculation

Based on the preliminary experiment, the incidence of POST associated with the use of a LMA was approximately 28%. It was hypothesized that administration of different doses of magnesium sulfate gargle would reduce the incidence of POST to 15% in the 10 mg/kg group, 10% in the 20 mg/kg group, and 5% in the 30 mg/kg group, respectively. Using PASS 15.0 software and the Chi-Square Test for Multiple Comparisons of Proportions, with a significance level (Type I error) set at 5% and statistical power of 90% (Type II error at 10%), the required total sample size was calculated to be 215 patients. To account for potential dropouts, an additional 10% of patients were included in each group. Consequently, the final sample size was determined as 60 patients per group, resulting in a total sample size of 240 patients.

### 2.7 Statistical analysis

The Kolmogorov-Smirnov test was employed to assess the normality of continuous variables. Normally distributed data are presented as mean ± standard deviation and were analyzed using one-way analysis of variance (ANOVA), with *post hoc* pairwise comparisons adjusted by the Bonferroni correction. For non-normally distributed continuous variables, the Kruskal–Wallis test was employed, with results expressed as median [interquartile range; IQR], and *post hoc* pairwise comparisons were conducted using the Dunn test. Categorical variables are reported as percentages and were analyzed using the chi-square test. When a significant overall difference among groups was observed, pairwise comparisons were conducted using the chi-square test. The Cochran-Armitage trend test was employed to evaluate the trend in the incidence of POST as the dosage of magnesium sulfate gargle increased in the PACU and at 2, 6, and 24 h postoperatively. Probit regression analysis was conducted to estimate the ED90 values of magnesium sulfate gargle employed for preventing POST in the PACU and at 2 h postoperatively. A *P*-value <0.05 was considered statistically significant. All statistical analyses were carried out using SPSS version 25.0 (IBM SPSS, Inc., Chicago, IL, United States).

## 3 Results

**FIGURE 1:**
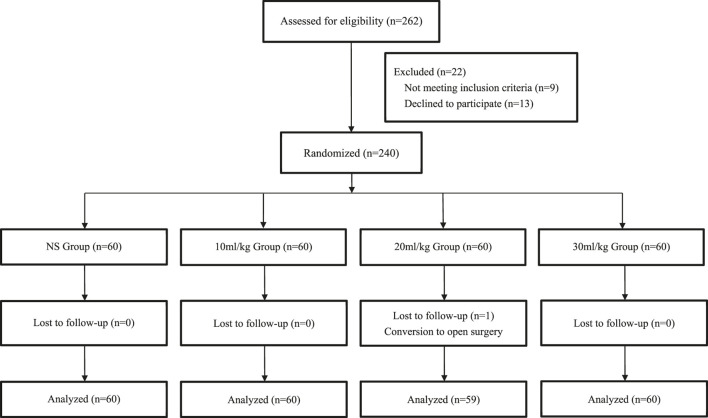
Study flow diagram.

This study excluded 22 patients and enrolled 240 patients who underwent random intervention. Among these, one patient from the 20 mg/kg group was excluded due to conversion from laparoscopic to open surgery. Ultimately, 239 patients were included in the final analysis: 60 in the NS group, 60 in the 10 mg/kg group, 59 in the 20 mg/kg group, and 60 in the 30 mg/kg group. The patient enrollment flowchart is presented in Figure 1. No statistically significant differences were observed in baseline characteristics, comorbidities, or types of surgery across the groups (*P*_all_ > 0.05), as summarized in Table 1.

**TABLE 1:** Baseline characteristics.

Characteristics	NS group (n = 60)	10 mg/kg group (n = 60)	20 mg/kg group (n = 59)	30 mg/kg group (n = 60)	*P* Value
Age, year	44.0 ± 9.8	45.1 ± 10.6	47.7 ± 10.5	44.0 ± 9.4	0.147
BMI, kg/m^2^	24.9 ± 3.8	24.6 ± 2.7	23.7 ± 3.1	23.9 ± 2.9	0.135
Comorbidity, n (%)					
Hypertension	8 (13.3%)	9 (15.0%)	9 (15.3%)	6 (10.0%)	0.824
Diabetes	0 (0.0%)	1 (1.7%)	2 (3.4%)	0 (0.0%)	0.287
Coronary heart disease	0 (0.0%)	1 (1.7%)	1 (1.7%)	0 (0.0%)	0.565
Type of surgery, n (%)					
Hysterectomy	23 (38.3%)	26 (43.3%)	26 (44.1%)	25 (41.7%)	0.923
Ovarian surgery	16 (26.7%)	18 (30.0%)	15 (25.4%)	16 (26.7%)	0.951
Myomectomy	19 (31.7%)	14 (23.3%)	16 (27.1%)	18 (30.0%)	0.756
Other	2 (3.3%)	2 (3.3%)	2 (3.4%)	1 (1.7%)	0.930
Mallampati classification (III-IV), n (%)	1 (1.7%)	2 (3.3%)	2 (3.4%)	2 (3.3%)	0.930

Data are presented as mean ± SD, and number (%).

The incidence and severity of POST assessed at four time points: in the PACU, and 2, 6, and 24 h postoperatively, as detailed in Table 2. As the dosage of magnesium sulfate gargle increased across different observation time points (in the PACU and at 2, 6, and 24 h postoperatively), the incidence of POST decreased (PACU, *P* = 0.002; 2 h postoperatively, *P* = 0.002; 6 h postoperatively, *P* < 0.001; 24 h postoperatively, *P* = 0.012), showing a consistent downward trend, with *P* values from the Cochran - Armitage test being <0.001, = 0.001, <0.001, and = 0.004 respectively. Compared with the NS group, the 30 mg/kg group showed reduced POST incidence in the PACU (6.7% vs. 31.7%, *P* < 0.001), as well as at 2 h (6.7% vs. 28.3%, *P* = 0.002), 6 h (3.3% vs. 23.3%, *P* = 0.001), and 24 h (1.7% vs. 15.0%, *P* = 0.008) postoperatively. The 20 mg/kg group showed reduced POST incidence at 2 h (6.8% vs. 28.3%, *P* = 0.002) and 6 h (3.4% vs. 23.3%, *P* = 0.001) postoperatively. However, no statistically significant differences were observed in the severity of POST across groups at any of the measured time points (*P*_all_ > 0.05).

**TABLE 2:** Incidence and intensity of postoperative sore throat.

Incidence and intensity	NS group (n = 60)	10 mg/kg group (n = 60)	20 mg/kg group (n = 59)	30 mg/kg group (n = 60)	*P* Value
Post-anesthesia care unit (PACU)					
Incidence, n (%)	19 (31.7%)^*^	11 (18.3%)	7 (11.9%)	4 (6.7%)	0.002
Intensity	1 [1, 2]	1 [1, 1]	1 [1, 2]	2 [2, 2]	0.056
2 h postopertively					
Incidence, n (%)	17 (28.3%)^#^	9 (15.0%)	4 (6.8%)	4 (6.7%)	0.002
Intensity	1 [1, 2]	1 [1, 2]	1 [1, 1]	1 [1, 1]	0.540
6 h postopertively					
Incidence, n (%)	14 (23.3%)^†^	6 (10.0%)	2 (3.4%)	2 (3.3%)	<0.001
Intensity	1 [1, 1]	1 [1, 1]	1 [1, 1]	1 [1, 1]	0.882
24 h postopertively					
Incidence, n (%)	9 (15.0%)^‡^	3 (5.0%)	2 (3.4%)	1 (1.7%)	0.012
Intensity	1 [1, 2]	1 [1, 1]	1 [1, 1]	1 [1, 1]	0.813

Data are presented as number (%) and median [IQR].

^*^ *P* < 0.001 vs. 30 mg/kg group.

^#^ *P* = 0.002 vs. 30 mg/kg group; *P* = 0.002 vs. 20 mg/kg group.

^†^ *P* = 0.001 vs. 30 mg/kg group; *P* = 0.001 vs. 20 mg/kg group.

^‡^ *P* = 0.008 vs. 30 mg/kg group.

The results of the probit regression analysis revealed that the ED90 values of magnesium sulfate gargle for preventing POST in the PACU and at 2 h postoperatively were 22.747 mg/kg (95% CI: 16.524–37.616) and 18.828 mg/kg (95% CI: 12.847–31.396), respectively, with a Pearson chi-square goodness-of-fit test indicated *P* = 0.937 and *P* = 0.569. No statistically significant differences were observed in LMA ventilation-related parameters and adverse events across groups (*P*_all_ > 0.05), as presented in Table 3.

**TABLE 3:** Factors associated with laryngeal mask intubation and adverse effects.

Parameters	NS group (n = 60)	10 mg/kg group (n = 60)	20 mg/kg group (n = 59)	30 mg/kg group (n = 60)	*P* Value
Duration of intubation (sec)	92.5 [77.3, 120.8]	88.0 [63.3, 116.8]	95.0 [87.0, 123.0]	93.0 [78.3, 109.8]	0.121
LMA intubation time (sec)	5 [4, 7]	5 [4, 7]	5 [3, 7]	7 [4, 7]	0.737
LMA removal time (min)	3 [3, 3]	3 [3, 3]	3 [3, 3]	3 [3, 3]	0.142
Success on first attempt, n (%)	58 (96.7%)	58 (96.7%)	59 (100.0%)	60 (100.0%)	0.258
Intraoperative repositioning, n (%)	1 (1.7%)	2 (3.3%)	0 (0.0%)	1 (1.7%)	0.571
Conversion to tracheal intubation, n (%)	0 (0.0%)	0 (0.0%)	0 (0.0%)	0 (0.0%)	1.000
Oropharyngeal leak pressure (cmH_2_O)	25 [22, 27]	25 [23, 27]	25 [23, 27]	24 [23, 27]	0.940
Blood staining on the LMA	0 (0.0%)	1 (1.7%)	1 (1.7%)	0 (0.0%)	0.565
Cough, n (%)	0 (0.0%)	0 (0.0%)	0 (0.0%)	0 (0.0%)	1.000
Intraoperative hypoxemia (SpO_2_ < 90%), n (%)	1 (1.7%)	0 (0.0%)	0 (0.0%)	0 (0.0%)	0.392
Dysphagia, n (%)					
2 h postopertively	3 (5.0%)	3 (5.0%)	3 (5.1%)	3 (5.0%)	1.000
6 h postopertively	2 (3.3%)	1 (1.7%)	2 (3.4%)	2 (3.3%)	0.930
24 h postopertively	1 (1.7%)	0 (0.0%)	0 (0.0%)	1 (1.7%)	0.572
Hoarseness, n (%)					
2 h postopertively	2 (3.3%)	2 (3.3%)	1 (1.7%)	2 (3.3%)	0.936
6 h postopertively	1 (1.7%)	1 (1.7%)	1 (1.7%)	1 (1.7%)	1.000
24 h postopertively	1 (1.7%)	0 (0.0%)	0 (0.0%)	0 (0.0%)	0.392

Data are presented as median [IQR] and number (%). LMA, laryngeal mask airway.

## 4 Discussion

This study indicates that increasing the dosage of magnesium sulfate gargle is associated with a reduced incidence of POST in the PACU, and at 2, 6, and 24 h postoperatively. The estimated effective dose for preventing POST in 90% of patients (ED90) was determined to be 22.747 mg/kg (95% CI: 16.524–37.616) and 18.828 mg/kg (95% CI: 12.847–31.396) respectively, in the PACU and at 2 h postoperatively.

In this study, in the absence of preventive interventions, the incidence of POST in patients undergoing laparoscopic surgery with LMA were 31.7% in the PACU and 28.3% 2 h postoperatively, respectively—values closely comparable to the 29.8% reported by Lee et al. (2020) and the 29.4% reported by Moulder et al. (2016). According to existing literature, the reported incidence of POST ranges from 26.5% to 76.9%, with variations primarily attributed to differences in surgical techniques, patient positioning, types of airway management, materials used in airway devices, and the implementation of preventive measures (Teshome et al., 2023; van Esch et al., 2017). The primary mechanisms underlying POST involve mechanical stress induced by laryngoscope and endotracheal tube, as well as direct mucosal trauma caused by the tracheal tube cuff or the tube itself (L'Hermite et al., 2017).

Current evidence suggests that the use of LMA is associated with a significantly lower incidence and severity of POST compared to tracheal intubation. Van Esch et al. (2017) conducted a meta-analysis demonstrating that LMA not only maintains adequate ventilation but also reduces the risk of postoperative airway complications, including POST, dysphagia, and dysphonia, relative to tracheal intubation. Lee et al. (2020) compared the newer generation LMA (i-gel) with a tracheal tube-based device (air-Q) and reported a significantly higher airway leak pressure in the i-gel group than in the air-Q group (5.5 vs. 5.0 cm H_2_O, *P* < 0.001). However, no statistically significant differences were observed between the two groups in the incidence of POST (29.8% vs. 16.3%, *P* = 0.206), dysphagia (34.0% vs. 32.6%, *P* = 1.000), or dysphonia (34.0% vs. 27.9%, *P* = 0.689).

Magnesium sulfate is commonly used NMDA receptor antagonists. Owing to their analgesic and anti-inflammatory properties, they are widely utilized in clinical anesthesia and have shown potential in reducing the incidence and severity of POST. Borazan et al. (2012) demonstrated that administration of 610 mg magnesium citrate lozenges 30 min prior to surgery significantly reduced the incidence of POST at 2 and 4 h postoperatively, although no significant benefit was observed at 24 h. Similar results were also found in our study; specifically, the incidence of POST gradually declines over time. Furthermore, a meta-analysis indicated that magnesium sulfate is effective in preventing POST within 24 h following tracheal intubation, with an efficacy comparable to that of licorice lozenges and hormonal agents—odds ratios (OR) of 0.10 (95% CI: 0.03–0.26), 0.14 (95% CI: 0.03–0.55), and 0.11 (95% CI: 0.06–0.22), respectively (Singh et al., 2020). In our study, the incidence of POST was significantly reduced in patients who gargled with 10, 20, and 30 mg/kg of magnesium sulfate in the PACU and at 2, 6, and 24 h postoperatively. At each time point, the incidence of POST gradually decreased with the increase in the gargle dose. Among the gargled doses, 30 mg/kg demonstrated the greatest efficacy across all time points, while 20 mg/kg showed significant effectiveness at 2 and 6 h after surgery. These findings indicate that magnesium sulfate gargling is an effective intervention for reducing POST incidence, with both 20 mg/kg and 30 mg/kg representing effective dosage regimens. Singh et al. (2019) administered magnesium sulfate via mouthwash (20 mg/kg), lozenges (100 mg), or nebulization (225–500 mg) 15–30 min prior to surgery and reported a reduction in POST incidence from 24.5% to 7.2%, confirming its preventive potential following tracheal intubation. The incidence of POST observed with the 20 mg/kg dose in our study was consistent with these previously published results, with the range of 3.4%–6.8%. Furthermore, current literature lacks data on the ED90 of magnesium sulfate when used as a gargle for POST prevention. In our study, the ED90 of magnesium sulfate for preventing POST in patients undergoing general anesthesia with LMA was determined to be 22.747 mg/kg (95% CI: 16.524–37.616) and 18.828 mg/kg (95% CI: 12.847–31.396) respectively, in the PACU and at 2 h postoperatively. Based on these findings, we recommend 20 mg/kg magnesium sulfate gargl as an optimal dose for preventing POST following LMA ventilation, which provides clinical practice implications for the use of magnesium sulfate gargle and the selection of dosage.

In this study, all patients demonstrated a high success rate of initial intubation and satisfactory OLP, accompanied by a low incidence of intraoperative repositionings and conversion to endotracheal intubation. Among the observed airway complications, the incidence of blood staining on the LMA, hoarseness, and hypoxemia were relatively low. The incidences of dysphagia (primarily attributable to POST) and hoarseness did not exceed 5% and approximately 3%, respectively, at any measured time point, complications that may be associated with LMA compression. All patients fully recovered approximately 24 h postoperatively, indicating that the use of the LMA in gynecological laparoscopic surgery is safe and consistent with current clinical practice.

This study has several limitations. First, it excluded surgical procedures with durations of less than 1 hour or more than 4 hours, and because long-term (e.g., >120 min) application of laryngeal mask ventilation may increase the incidence of adverse events and impact ventilation effect, this may limit the generalizability of the findings. Second, blood magnesium levels were not measured, thereby restricting a more comprehensive assessment of the safety profile of magnesium sulfate gargle. Although the maximum dose of magnesium sulfate gargle administered in this study (30 mg/kg) is supported by existing literature (Park et al., 2015) and has not been associated with significant safety concerns, a higher dose was not explored to avoid potential adverse effects related to magnesium sulfate administration.

In conclusion, the prophylactic administration of magnesium sulfate gargle can effectively reduce the incidence of POST following laryngeal mask airway (LMA) ventilation under general anesthesia. A dose of 20 mg/kg of magnesium sulfate gargle is considered optimal for preventing POST in patients undergoing gynecological laparoscopic surgery.

## Data Availability

The original contributions presented in the study are included in the article/supplementary material, further inquiries can be directed to the corresponding author.
